# Supplementary material for: Seasonal regulation of herbivory and nutrient effects on macroalgal recruitment and succession in a Florida coral reef
Source: PeerJ. 2016 Nov 2;4:e2643. doi: 10.7717/peerj.2643 (PMC5101614; doi:10.7717/peerj.2643)
Supplement: Data S4 [file peerj-04-2643-s004.docx]

**Fish communities, raw data description**

| **Variable** | **Description** | **Unit** |
| --- | --- | --- |
| Zone | Florida Keys |  |
| Site | Study area |  |
| Season | Season |  |
| Month | Month |  |
| Year | Year |  |
| Transects | 1 through 12 | 100m^2 |
| TotalDens | Density of fish | Ind/100m^2 |
| TotalBiom | Biomass of fish | g/100m^2 |
| HerbDens | Density of herbivorous fish | Ind/100m^2 |
| HerbBiom | Biomass of herbivoroou fish | g/100m^2 |

**Recruitment tiles, raw data description**

| **Variable** | **Description** | **Unit** |
| --- | --- | --- |
| Season | Season |  |
| Treatment | NE (Nutrient enrichment-Exclosure), AE (Ambient nutrient-Exclosure), NH (Nutrient enrichment-Uncaged), AH (Ambient nutrient-Uncaged) |  |
| Herbivory | E (Exclosure), H (Uncaged) |  |
| Nutrient | N(Nutrient enrichment), A (Ambient nutrient) |  |
| Ceramium clavulatum | Percent cover | % |
| Ceramiun nitens | Percent cover | % |
| Cladophora catenata | Percent cover | % |
| Derbesia sp. | Percent cover | % |
| Enteromorpha chaetomorphoides | Percent cover | % |
| Spyridia clavata | Percent cover | % |
| Gelidiopsis intricata | Percent cover | % |
| Gelidium sp. | Percent cover | % |
| Gellidiela acerosa | Percent cover | % |
| Neomeris annulata | Percent cover | % |
| Plenosporium flexuosum | Percent cover | % |
| Stypopodium zonale | Percent cover | % |
| Ulva sp | Percent cover | % |
| Valonia macrophysa | Percent cover | % |
| Champia sp. | Percent cover | % |
| Neomeris sp. | Percent cover | % |
| Cladophora laetevirens | Percent cover | % |
| Dictiosphaeria cavernosa | Percent cover | % |
| Dictyota menstrualis | Percent cover | % |
| Gracilariopsis lemaneiformes | Percent cover | % |
| Rhodimenia pseudopalmata | Percent cover | % |
| Ulva flexuosa | Percent cover | % |
| Chondria aff. Polyrhisa | Percent cover | % |
| Cladophora catenata | Percent cover | % |
| Amphiroa sp | Percent cover | % |
| Ceramium curniculatum | Percent cover | % |
| Chondria aff. Leptocremon | Percent cover | % |
| Gelidiopsis planicaulis | Percent cover | % |
| Turbinaria sp. | Percent cover | % |
| Apoglossum rustifolium | Percent cover | % |
| Chodria sp. 1 | Percent cover | % |
| Cladophora prolifera | Percent cover | % |
| Griffithsia sp | Percent cover | % |
| Derbesia osterhoutii (Halisysis stage) | Percent cover | % |
| Hildenbrandia rubra | Percent cover | % |
| Ventricaria venticosa | Percent cover | % |
| Laurencia poiteaui | Percent cover | % |
| Acetabularia myriospora | Percent cover | % |
| Pterocladiella capillacea | Percent cover | % |
| Griffithsia globulifera | Percent cover | % |
| Enteromorpha prolifera | Percent cover | % |
| Meristiella schrammii | Percent cover | % |
| Acetabularia sp | Percent cover | % |
| Amphiroa brasiliana | Percent cover | % |
| Asparagopsis taxiformis (falkenbergia) | Percent cover | % |
| Sphacelaria sp. | Percent cover | % |
| Ceramiun cimbricum | Percent cover | % |
| Chrysymenia ventricosa | Percent cover | % |
| Polysiphonia atlantica | Percent cover | % |
| Laurencia sp.3 | Percent cover | % |
| Amphiroa tribulus | Percent cover | % |
| Laurencia sp. 2 | Percent cover | % |
| Hypnea sp. | Percent cover | % |
| Polysiphonia sp. 2 | Percent cover | % |
| Champia parvula | Percent cover | % |
| Dictyota cervicornis | Percent cover | % |
| Amphiroa rigida | Percent cover | % |
| Gelidiopsis sp. | Percent cover | % |
| Gelidium americanum | Percent cover | % |
| Sphacelaria tribuloides | Percent cover | % |
| Ceramium flaccidum | Percent cover | % |
| Cladophora albida | Percent cover | % |
| Jania sp | Percent cover | % |
| Polysiphonia sp. 3 | Percent cover | % |
| Centroceras clavulatum | Percent cover | % |
| Wurdemannia miniata | Percent cover | % |
| Gelidiella sp. | Percent cover | % |
| Chaetomorpha sp. | Percent cover | % |
| Heterosiphonia sp | Percent cover | % |
| Fermania sp. | Percent cover | % |
| Dictyota pulchella | Percent cover | % |
| Acetabularia pusila | Percent cover | % |
| Cladophora sp | Percent cover | % |
| Herposiphonia secunda | Percent cover | % |
| Derbesia marina | Percent cover | % |
| Enteromorpha sp. | Percent cover | % |
| Gelidiella sanctarum | Percent cover | % |
| Polysiphonia sp.1 | Percent cover | % |
| Laurencia intricata | Percent cover | % |
| Bryobesia johanne | Percent cover | % |
| Polysiphonia scopulorum | Percent cover | % |
| Sargassum sp. | Percent cover | % |
| Dictyota sp. | Percent cover | % |
| Hypnea valentiae | Percent cover | % |
| Heterosiphonia gibbesi | Percent cover | % |
| Laurencia sp. 1 | Percent cover | % |
| Jania adhaerens | Percent cover | % |
| Ectocarpus sp. | Percent cover | % |
| Neosiphonia howei | Percent cover | % |
| Amphiroa fragilissima | Percent cover | % |
| Hypnea spinella | Percent cover | % |
| Laurencia cervicornis | Percent cover | % |
| Jania capillacea | Percent cover | % |
| Peyssonelia sp. | Percent cover | % |
| CCA | Percent cover | % |
| Cyanobacteria | Percent cover | % |

**Succession tiles, raw data description**

| **Variable** | **Description** | **Unit** |
| --- | --- | --- |
| Season | Season |  |
| Treatment | NE (Nutrient enrichment-Exclosure), AE (Ambient nutrient-Exclosure), NH (Nutrient enrichment-Uncaged), AH (Ambient nutrient-Uncaged) |  |
| Herb | E (Exclosure), H (Uncaged) |  |
| Nut | N(Nutrient enrichment), A (Ambient nutrient) |  |
| Filament | Percent cover | % |
| Foliose | Percent cover | % |
| Leathery | Percent cover | % |
| Art-Calc | Percent cover | % |
| Crustose | Percent cover | % |
| Total | Percent cover | % |

**Established communities, raw data description**

| **Variable** | **Description** | **Unit** |
| --- | --- | --- |
| Season | Season |  |
| Treatment | NE (Nutrient enrichment-Exclosure), AE (Ambient nutrient-Exclosure), NH (Nutrient enrichment-Uncaged), AH (Ambient nutrient-Uncaged) |  |
| Herb | E (Exclosure), H (Uncaged) |  |
| Nut | N(Nutrient enrichment), A (Ambient nutrient) |  |
| Filament | Percent cover | % |
| Foliose | Percent cover | % |
| Leathery | Percent cover | % |
| Art-Calc | Percent cover | % |
| Crustose | Percent cover | % |
| Total | Percent cover | % |
