# Supplementary material for: Seasonal regulation of herbivory and nutrient effects on macroalgal recruitment and succession in a Florida coral reef
Source: PeerJ. 2016 Nov 2;4:e2643. doi: 10.7717/peerj.2643 (PMC5101614; doi:10.7717/peerj.2643)
Supplement: Appendix S1 [file peerj-04-2643-s006.docx]

Dissolved Inorganic Nitrogen (DIN), Soluble Reactive Phosphorus (SRP) and water temperature from Molasses Reef (approximately 3 Km from the study site) before, during and after our study period. Dash line indicates our study period.

Data provided by the SERC-FIU Water Quality Monitoring Network. Supported by EPA Agreement #X7 00D02412-1 and NOAA Agreement #NA09NOS4260253.’

<http://serc.fiu.edu/wqmnetwork/FKNMS-CD/DataDL.htm>
