# Supplementary material for: Seasonal regulation of herbivory and nutrient effects on macroalgal recruitment and succession in a Florida coral reef
Source: PeerJ. 2016 Nov 2;4:e2643. doi: 10.7717/peerj.2643 (PMC5101614; doi:10.7717/peerj.2643)
Supplement: Appendix S2 [file peerj-04-2643-s007.docx]

Appendix I. List of algal species found in the study and their assigned form functional group

| **List of algal species by season of Pickles reef (field and laboratory observations) S=101** | | | | | | | | | | | |
| --- | --- | --- | --- | --- | --- | --- | --- | --- | --- | --- | --- |
| **Red** | | **FFG** | **Fall** | **Winter** | **Spring** | **Brown** | | **FFG** | **Fall** | **Winter** | **Spring** |
| 1 | *Amphiroa brasiliana*** | 6 | 0 | 0 | 1 | 1 | *Dictyota cervicornis* | 3.5 | 0 | 1 | 1 |
| 2 | ***Amphiroa fragilissima*** | **6** | **1** | **1** | **1** | 2 | *Dictyota menstrualis* | 3.5 | 0 | 0 | 1 |
| 3 | ***Amphiroa rigida*** | **6** | **1** | **1** | **1** | 3 | *Dictyota pulchella* | 3.5 | 0 | 1 | 1 |
| 4 | *Amphiroa* sp.** | 6 | 1 | 0 | 0 | 4 | ***Dictyota* sp.** | **3.5** | **1** | **1** | **1** |
| 5 | ***Amphiroa tribulus*** | **6** | **1** | **1** | **1** | 5 | *Ectocarpus* sp*.* | 2 | 0 | 1 | 0 |
| 6 | *Apoglossum rustifolium*** | 3 | 0 | 0 | 1 | 6 | *Fermania* sp*.* | 2 | 0 | 1 | 0 |
| 7 | *Asparagopsis taxiformis (falkenbergia)* | 2 | 0 | 1 | 1 | 7 | ***Sargassum* sp*.*** | **5** | **1** | **1** | **1** |
| 8 | *Ceramium clavulatum*** | 2 | 0 | 0 | 1 | 8 | *Sphacelaria* sp*.* | 2 | 0 | 1 | 0 |
| 9 | *Ceramium curniculatum*** | 2 | 0 | 0 | 1 | 9 | *Sphacelaria tribuloides* | 2 | 0 | 0 | 1 |
| 10 | *Ceramium flaccidum*** | 2 | 1 | 0 | 1 | 10 | *Stypopodium zonale* | 3.5 | 0 | 0 | 1 |
| 11 | *Ceramiun cimbricum*** | 2 | 0 | 0 | 1 | 11 | *Turbinaria turbinata* | 5 | 1 | 0 | 0 |
| 12 | *Ceramiun nitens*** | 2 | 0 | 1 | 0 |  | **Total number of species** |  | 3 | 7 | 7 |
| 13 | *Champia parvula*** | 2 | 0 | 1 | 1 | **Green** | |  |  | | |
| 14 | *Champia* sp.** | 2 | 0 | 1 | 0 | 1 | *Acetabularia myriospora* | 6 | 0 | 1 | 1 |
| 15 | *Chodria* sp. 1** | 4 | 0 | 0 | 1 | 2 | ***Acetabularia pusilla*** | **6** | **1** | **1** | **1** |
| 16 | *Chodria* sp. 2 *(aff. Polyrhisa)* | 4 | 0 | 1 | 0 | 3 | *Acetabularia* sp.** | 6 | 0 | 0 | 1 |
| 17 | *Chondria* sp. 3 *(aff. Leptocremon)* | 4 | 0 | 1 | 0 | 4 | *Bryobesia johanne* | 2 | 1 | 1 | 1 |
| 18 | *Chrysymenia ventricosa*** | 4 | 0 | 0 | 1 | 5 | *Chaetomorpha* sp*.* | 2 | 0 | 1 | 0 |
| 19 | *Gelidiella acerosa*** | 4 | 1 | 0 | 0 | 6 | *Cladophora albida* | 2 | 1 | 1 | 0 |
| 20 | *Gelidiella sanctarum*** | 4 | 0 | 1 | 1 | 7 | *Cladophora catenata*** | 2 | 1 | 0 | 1 |
| 21 | *Gelidiella* sp*.* | 4 | 0 | 1 | 0 | 8 | *Cladophora laetevirens* | 2 | 0 | 1 | 0 |
| 22 | *Gelidiopsis intricata*** | 4 | 0 | 0 | 1 | 9 | *Cladophora prolifera* | 2 | 0 | 1 | 1 |
| 23 | *Gelidiopsis planicaulis* | 4 | 0 | 1 | 0 | 10 | *Cladophora* sp. | 2 | 1 | 0 | 0 |
| 24 | *Gelidiopsis* sp. | 4 | 0 | 1 | 0 | 11 | *Derbesia marina* | 2 | 1 | 1 | 1 |
| 25 | *Gelidium americanum*** | 4 | 1 | 0 | 0 | 12 | *Derbesia osterhoutii (Halisysis* stage*)* | 2 | 0 | 1 | 0 |
| 26 | *Gelidium* sp*.*** | 4 | 0 | 1 | 0 | 13 | *Derbesia* sp*.*** | 2 | 0 | 1 | 0 |
| 27 | *Gracilariopsis lemaneiformes*** | 4 | 0 | 1 | 0 | 14 | *Dictyosphaeria cavernosa* | 3 | 0 | 1 | 0 |
| 28 | *Griffithsia globulifera*** | 2 | 0 | 0 | 1 | 15 | *Enteromorpha chaetomorphoides*** | 2 | 1 | 0 | 0 |
| 29 | *Griffithsia* sp.** | 2 | 0 | 1 | 1 | 16 | *Enteromorpha prolifera*** | 2 | 0 | 0 | 1 |
| 30 | *Herposiphonia secunda* | 2 | 0 | 1 | 0 | 17 | *Enteromorpha* sp. | 2 | 1 | 1 | 0 |
| 31 | ***Herposiphonia* sp.**** | **2** | **1** | **1** | **1** | 18 | *Halimeda opuntia ** | 6 | 0 | 0 | 0 |
| 32 | ***Heterosiphonia gibbesi*** | **2** | **1** | **1** | **1** | 19 | *Halimeda tuna ** | 6 | 0 | 0 | 0 |
| 33 | *Heterosiphonia* sp.** | 2 | 0 | 1 | 0 | 20 | *Neomeris* sp.* | 5 | 1 | 0 | 0 |
| 34 | *Hildenbrandia rubra*** | 7 | 0 | 0 | 1 | 21 | *Neomeris annulata*** | 5 | 0 | 0 | 1 |
| 35 | *Hypnea* sp*.*** | 4 | 1 | 1 | 0 | 22 | *Penicillus capitatus ** | 6 | 0 | 0 | 0 |
| 36 | ***Hypnea spinella*** | **4** | **1** | **1** | **1** | 23 | *Ulva flexuosa*** | 3 | 0 | 0 | 1 |
| 37 | ***Hypnea valentiae***** | **4** | **1** | **1** | **1** | 24 | *Ulva* sp.** | 3 | 1 | 0 | 0 |
| 38 | ***Jania adhaerens*** | **6** | **1** | **1** | **1** | 25 | *Valonia macrophysa* | 3 | 0 | 1 | 0 |
| 39 | ***Jania capillacea*** | **6** | **1** | **1** | **1** | 26 | *Ventricaria ventricosa* | 3 | 0 | 1 | 1 |
| 40 | ***Jania* sp.**** | **6** | **1** | **1** | **1** | **Total number of species** | |  | 10 | 14 | 11 |
| 41 | ***Laurencia cervicornis*** | **4** | **1** | **1** | **1** | **Blue-green algae** | |  |  | | |
| 42 | *Laurencia intricata*** | 4 | 0 | 0 | 1 | 1 | *Lynbya* sp. ** | 1 | N/A | N/A | N/A |
| 43 | *Laurencia poiteaui*** | 4 | 0 | 0 | 1 | 2 | *Symploca* sp*. *** | 1 | N/A | N/A | N/A |
| 44 | ***Laurencia* sp. *1*** | **4** | **1** | **1** | **1** | 3 | *Calothrix* sp*. *** | 1 | N/A | N/A | N/A |
| 45 | *Laurencia* sp. *2* | 4 | 0 | 1 | 0 | 4 | *Rivularia* sp. ** | 1 | N/A | N/A | N/A |
| 46 | *Laurencia* sp. *3*** | 4 | 0 | 0 | 1 | **Total number of species** | |  | 1 | 1 | 1 |
| 47 | *Meristiella schrammii*** | 4 | 0 | 0 | 1 |  |  |  |  |  |  |
| 48 | ***Neosiphonia howei*** | **2** | **1** | **1** | **1** |  | **FFG-Steneck and Dethier form-functional groups (1994)** | | | | |
| 50 | *Plenosporium flexuosum*** | 2 | 0 | 1 | 0 |  | 1- Microalgae | | | | |
| 51 | *Polysiphonia atlantica* | 2 | 0 | 1 | 0 |  | 2- Filamentous algae | | | | |
| 52 | *Polysiphonia scopulorum*** | 2 | 0 | 0 | 1 |  | 3- Foliose algae | | | | |
| 53 | *Polysiphonia* sp. 2 | 2 | 0 | 1 | 0 |  | 3.5- Corticated foliose | | | | |
| 54 | *Polysiphonia* sp. 3 | 2 | 0 | 1 | 0 |  | 4- Corticated macrophytes | | | | |
| 55 | *Polysiphonia* sp.1 | 2 | 1 | 1 | 0 |  | 5- Leathery macrophytes | | | | |
| 56 | *Pterocladiella capillacea*** | 4 | 0 | 0 | 1 |  | 6- Articulates calcareous algae | | | | |
| 57 | *Rhodimenia pseudopalmata*** | 2 | 0 | 0 | 1 |  | 7- Crustose algae | | | | |
| 58 | *Spyridia clavata ** | 2 | 0 | 0 | 1 |  | * Species not recluted on tiles n = 5 | | | | |
| 59 | *Wurdemannia miniata*** | 4 | 0 | 0 | 1 |  | ** Species identified only on tiles n = 46 | | | | |
| 60 | **Crustose Coraline Algae** | **7** | **1** | **1** | **1** |  | Bold species name were present in all seasons | | | | |
| **Total number of species** | |  | 20 | 36 | 38 |  |  |  |  |  |  |
