# Supplementary material for: Seasonal regulation of herbivory and nutrient effects on macroalgal recruitment and succession in a Florida coral reef
Source: PeerJ. 2016 Nov 2;4:e2643. doi: 10.7717/peerj.2643 (PMC5101614; doi:10.7717/peerj.2643)
Supplement: Appendix S3 [file peerj-04-2643-s008.docx]

Appendix II. Statistical Analysis

FISH COMMUNITY

One-way ANOVA. Total biomass

Df Sum Sq Mean Sq F value Pr(>F)

Month 3 8983146 2994382 0.23 0.875

Residuals 44 573492192 13033913

One-way ANOVA. Total density

Df Sum Sq Mean Sq F value Pr(>F)

Month 3 694 231.5 0.449 0.719

Residuals 44 22677 515.4

One-way ANOVA. Herbivore biomass

Df Sum Sq Mean Sq F value Pr(>F)

Month 3 14819142 4939714 0.303 0.823

Residuals 44 716903155 16293254

One-way ANOVA. Herbivore density

Df Sum Sq Mean Sq F value Pr(>F)

Month 3 476 158.8 0.745 0.531

Residuals 44 9378 213.1

RECRUITMENT TILES

Richness of recruitment tiles. Split plot ANOVA

Value Std.Error DF t-value p-value

(Intercept) 8.500000 1.825595 22 4.656016 0.0001

HerbivoryH 2.250000 2.325814 8 0.967403 0.3617

NutrientN 2.250000 2.325814 8 0.967403 0.3617

SeasonSpring 7.750000 2.322214 22 3.337333 0.0030***

SeasonWinter 2.500000 2.322214 22 1.076559 0.2933

HerbivoryH:NutrientN -4.602093 3.433429 8 -1.340378 0.2169

HerbivoryH:SeasonSpring -3.250000 3.284106 22 -0.989615 0.3331

HerbivoryH:SeasonWinter 0.250000 3.284106 22 0.076124 0.9400

NutrientN:SeasonSpring -5.750000 3.284106 22 -1.750857 0.0939

NutrientN:SeasonWinter -1.250000 3.284106 22 -0.380621 0.7071

HerbivoryH:NutrientN:SeasonSpring 5.583333 4.834073 22 1.154996 0.2605

HerbivoryH:NutrientN:SeasonWinter 1.833333 4.834073 22 0.379252 0.7081

Total abundance of recruitment tiles across seasons. Split plot ANOVA

Value Std.Error DF t-value p-value

(Intercept) 48.37500 12.27125 22 3.942142 0.0007

HerbivoryH 18.67500 17.35416 8 1.076111 0.3133

NutrientN 25.13750 17.35416 8 1.448500 0.1855

SeasonSpring 44.90000 17.35416 22 2.587275 0.0168*

SeasonWinter -0.70000 17.35416 22 -0.040336 0.9682

HerbivoryH:NutrientN -44.10417 25.54464 8 -1.726553 0.1225

HerbivoryH:SeasonSpring -1.02500 24.54249 22 -0.041764 0.9671

HerbivoryH:SeasonWinter -31.86250 24.54249 22 -1.298258 0.2076

NutrientN:SeasonSpring 44.02500 24.54249 22 1.793828 0.0866

NutrientN:SeasonWinter 7.37500 24.54249 22 0.300499 0.7666

HerbivoryH:NutrientN:SeasonSpring -44.25000 36.12557 22 -1.224894 0.2336

HerbivoryH:NutrientN:SeasonWinter 19.50417 36.12557 22 0.539899 0.5947

Tukey multiple comparisons of means for total abundance of macroalgae across seasons (95 % family-wise confidence interval)

Seasons diff lwr upr p adj

Fall-Winter 8.226667 -13.76338 30.21672 0.6329512

**Spring**-Winter 64.343333 42.35328 86.33338 0.0000001 ***

**Spring**-Fall 56.116667 34.12662 78.10672 0.0000013 ***

Tukey multiple comparisons of means for total abundance of macroalgae interactions of herbivores and nutrient (95 % family-wise confidence interval)

Interactions Herbivory:Nutrient diff lwr upr p adj

Exclosure:Ambient - Uncaged:Enrichment 2.37 -26.90 31.64 0.996

Uncaged:Ambient - Uncaged:Enrichment 10.08 -19.19 39.36 0.788

**Exclosure:Enrichment** - Uncaged:Enrichment 44.64 15.37 73.91 0.001***

Uncaged:Ambient - Exclosure:Ambient 7.71 -19.39 34.81 0.867

**Exclosure:Enrichment** - Exclosure:Ambient 42.27 15.17 69.37 0.001***

**Exclosure:Enrichment** - Uncaged:Ambient 34.55 7.46 61.66 0.008**

Permanova analysis of algal communities on recruitment tiles across seasons (species level)

Df SumsOfSqs MeanSqs F.Model R2 Pr(>F)

Herbivory 1 0.8199 0.81990 3.9406 0.06137 0.01 **

Nutrient 1 0.3340 0.33397 1.6051 0.02500 0.08

Season 2 3.1941 1.59707 7.6759 0.23908 0.01 **

Herbivory:Nutrient 1 0.3044 0.30443 1.4632 0.02279 0.16

Herbivory:Season 2 0.8937 0.44683 2.1476 0.06689 0.01 **

Nutrient:Season 2 0.4174 0.20870 1.0031 0.03124 0.43

Herbivory:Nutrient:Season 2 0.5307 0.26535 1.2753 0.03972 0.18

Residuals 33 6.8661 0.20806 0.51392

Total 44 13.3603 1.00000

Permanova analysis of algal communities on recruitment tiles in Fall (species level)

Df SumsOfSqs MeanSqs F.Model R2 Pr(>F)

Herbivory 1 0.27962 0.27962 1.5277 0.10097 0.07

Nutrient 1 0.22943 0.22943 1.2535 0.08285 0.17

Herbivory:Nutrient 1 0.24683 0.24683 1.3486 0.08913 0.20

Residuals 11 2.01332 0.18303 0.72704

Total 14 2.76921 1.00000

Permanova analysis of algal communities on recruitment tiles in Winter (species level)

Df SumsOfSqs MeanSqs F.Model R2 Pr(>F)

Herbivory 1 0.5747 0.57473 1.90534 0.13714 0.06

Nutrient 1 0.0923 0.09235 0.30615 0.02204 1.00

Herbivory:Nutrient 1 0.2056 0.20563 0.68170 0.04907 0.73

Residuals 11 3.3180 0.30164 0.79175

Total 14 4.1907 1.00000

Permanova analysis of algal communities on recruitment tiles in Spring (species level)

Df SumsOfSqs MeanSqs F.Model R2 Pr(>F)

Herbivory 1 0.8592 0.85922 6.1582 0.26798 0.01 **

Nutrient 1 0.4296 0.42959 3.0790 0.13399 0.04 *

Herbivory:Nutrient 1 0.3827 0.38267 2.7427 0.11935 0.04 *

Residuals 11 1.5348 0.13952 0.47868

Total 14 3.2062 1.00000

SUCCESSION TILES

Overall abundance of algae on succession tiles. Split plot ANOVA

Value Std.Error DF t-value p-value

(Intercept) 85.24251 14.13934 11 6.028749 0.0001

HerbivoryH -47.36751 18.29596 8 -2.588960 0.0322*

NutrientN -15.86751 18.29596 8 -0.867269 0.4111

SeasonJune -1.16667 19.50192 11 -0.059823 0.9534

HerbivoryH:NutrientN 1.11751 24.89951 8 0.044881 0.9653

HerbivoryH:SeasonJune 16.79167 25.79862 11 0.650875 0.5285

NutrientN:SeasonJune 3.79167 25.79862 11 0.146972 0.8858

HerbivoryH:NutrientN:SeasonJune 6.33333 35.15759 11 0.180141 0.8603

Abundance of filamentous algae on succession tiles. Split plot ANOVA

Value Std.Error DF t-value p-value

(Intercept) 53.33333 9.180293 11 5.809546 0.0001

HerbivoryH -33.95833 12.144386 8 -2.796217 0.0233*

NutrientN -48.95833 12.144386 8 -4.031355 0.0038**

SeasonJune -24.66667 12.982895 11 -1.899936 0.0840

HerbivoryH:NutrientN 38.95833 16.550008 8 2.353977 0.0464*

HerbivoryH:SeasonJune 57.41667 17.174755 11 3.343085 0.0066**

NutrientN:SeasonJune 44.04167 17.174755 11 2.564326 0.0263*

HerbivoryH:NutrientN:SeasonJune -56.66667 23.405246 11 -2.421110 0.0339*

Abundance of foliose algae on succession tiles. Split plot ANOVA

Value Std.Error DF t-value p-value

(Intercept) 33.33333 7.010026 11 4.755094 0.0006

HerbivoryH -24.20833 9.273393 8 -2.610515 0.0311*

NutrientN -18.20833 9.273393 8 -1.963503 0.0852

SeasonJune 0.00000 9.913674 11 0.000000 1.0000

HerbivoryH:NutrientN 19.70833 12.637504 8 1.559511 0.1575

HerbivoryH:SeasonJune -9.00000 13.114558 11 -0.686260 0.5068

NutrientN:SeasonJune -12.75000 13.114558 11 -0.972202 0.3518

HerbivoryH:NutrientN:SeasonJune 18.62500 17.872130 11 1.042125 0.3197

Abundance of leathery algae on succession tiles. Split plot ANOVA

Value Std.Error DF t-value p-value

(Intercept) 0.00000 6.409677 11 0.0000000 1.0000

HerbivoryH 0.62500 8.479206 8 0.0737097 0.9431

NutrientN 5.00000 8.479206 8 0.5896778 0.5717

SeasonJune 16.83333 7.832850 11 2.1490688 0.0547

HerbivoryH:NutrientN -5.62500 11.555210 8 -0.4867934 0.6395

HerbivoryH:SeasonJune -16.83333 10.361886 11 -1.6245433 0.1325

NutrientN:SeasonJune -12.83333 10.361886 11 -1.2385132 0.2413

HerbivoryH:NutrientN:SeasonJune 12.83333 14.120871 11 0.9088203 0.3829

Abundance of articulated calcareous algae on succession tiles. Split plot ANOVA

Value Std.Error DF t-value p-value

(Intercept) 0.00000 10.58612 11 0.0000000 1.0000

HerbivoryH 0.00000 14.00412 8 0.0000000 1.0000

NutrientN 42.50000 14.00412 8 3.0348206 0.0162**

SeasonJune 6.66667 14.78006 11 0.4510582 0.6607

HerbivoryH:NutrientN -42.50000 19.08440 8 -2.2269495 0.0566*

HerbivoryH:SeasonJune -6.66667 19.55218 11 -0.3409679 0.7396

NutrientN:SeasonJune -14.79167 19.55218 11 -0.7565226 0.4652

HerbivoryH:NutrientN:SeasonJune 14.79167 26.64513 11 0.5551358 0.5899

Abundance of crustose algae on succession tiles. Split plot ANOVA

Value Std.Error DF t-value p-value

(Intercept) -0.485724 4.188719 11 -0.1159601 0.9098

HerbivoryH 9.235724 5.362263 8 1.7223556 0.1233

NutrientN 2.360724 5.362263 8 0.4402477 0.6714

SeasonJune 0.000000 5.709905 11 0.0000000 1.0000

HerbivoryH:NutrientN -7.985724 7.294253 8 -1.0947967 0.3055

HerbivoryH:SeasonJune -8.125000 7.553494 11 -1.0756611 0.3051

NutrientN:SeasonJune 0.625000 7.553494 11 0.0827432 0.9355

HerbivoryH:NutrientN:SeasonJune 16.250000 10.293678 11 1.5786389 0.1427

Permanova analysis of community composition of succession tiles between seasons (FFG level)

Df SumsOfSqs MeanSqs F.Model R2 Pr(>F)

Herbivory 1 0.7723 0.77231 9.7369 0.20992 0.01 **

Nutrient 1 0.2713 0.27127 3.4201 0.07374 0.03 *

Season 1 0.2978 0.29783 3.7549 0.08096 0.01 **

Herbivory:Nutrient 1 0.1859 0.18593 2.3441 0.05054 0.07

Herbivory:Season 1 0.2250 0.22497 2.8364 0.06115 0.02 *

Nutrient:Season 1 0.0880 0.08800 1.1095 0.02392 0.34

Herbivory:Nutrient:Season 1 0.0937 0.09367 1.1810 0.02546 0.36

Residuals 22 1.7450 0.07932 0.47431

Total 29 3.6790 1.00000

Permanova analysis of community composition of succession tiles, January (FFG level)

Df SumsOfSqs MeanSqs F.Model R2 Pr(>F)

Herbivory 1 0.73047 0.73047 8.9575 0.36128 0.01 **

Nutrient 1 0.23144 0.23144 2.8381 0.11447 0.03 *

Herbivory:Nutrient 1 0.16293 0.16293 1.9979 0.08058 0.14

Residuals 11 0.89703 0.08155 0.44366

Total 14 2.02187 1.00000

Permanova analysis of community composition of succession tiles, June (FFG level)

Df SumsOfSqs MeanSqs F.Model R2 Pr(>F)

Herbivory 1 0.26681 0.266808 3.4612 0.19629 0.03 *

Nutrient 1 0.12783 0.127834 1.6583 0.09405 0.12

Herbivory:Nutrient 1 0.11667 0.116673 1.5135 0.08584 0.26

Residuals 11 0.84794 0.077086 0.62383

Total 14 1.35926 1.00000

ESTABLISHED COMMUNITIES

Overall algal abundance of established communities. Split plot ANOVA

Value Std.Error DF t-value p-value

(Intercept) 58.93402 9.192785 11 6.410899 0.0001

HerbivoryH -44.68402 11.879747 8 -3.761361 0.0055***

NutrientN 14.69098 11.879747 8 1.236641 0.2513

SeasonJune 27.83333 12.661083 11 2.198337 0.0502*

HerbivoryH:NutrientN -14.06598 16.166498 8 -0.870070 0.4096

HerbivoryH:SeasonJune 21.79167 16.749038 11 1.301070 0.2198

NutrientN:SeasonJune 18.79167 16.749038 11 1.121955 0.2858

HerbivoryH:NutrientN:SeasonJune -17.04167 22.825092 11 -0.746620 0.4709

Abundance of filamentous algae of established communities. Split plot ANOVA

Value Std.Error DF t-value p-value

(Intercept) 6.003670 5.894919 11 1.0184483 0.3303

HerbivoryH -4.003670 7.755623 8 -0.5162281 0.6197

NutrientN 1.746330 7.755623 8 0.2251695 0.8275

SeasonJune 18.166667 7.851445 11 2.3137992 0.0410*

HerbivoryH:NutrientN -3.371330 10.564912 8 -0.3191063 0.7578

HerbivoryH:SeasonJune 9.958333 10.386485 11 0.9587780 0.3583

NutrientN:SeasonJune 17.208333 10.386485 11 1.6568004 0.1258

HerbivoryH:NutrientN:SeasonJune -5.083333 14.154394 11 -0.3591347 0.7263

Abundance of foliose algae of established communities. Split plot ANOVA

Value Std.Error DF t-value p-value

(Intercept) 41.66667 8.781078 11 4.745052 0.0006

HerbivoryH -41.66667 11.616274 8 -3.586922 0.0071**

NutrientN 15.83333 11.616274 8 1.363030 0.2100

SeasonJune -8.00000 12.418318 11 -0.644210 0.5326

HerbivoryH:NutrientN -15.45833 15.830313 8 -0.976502 0.3574

HerbivoryH:SeasonJune 18.37500 16.427891 11 1.118525 0.2872

NutrientN:SeasonJune 7.37500 16.427891 11 0.448932 0.6622

HerbivoryH:NutrientN:SeasonJune -10.50000 22.387442 11 -0.469013 0.6482

Abundance of leathery algae of established communities. Split plot ANOVA

Value Std.Error DF t-value p-value

(Intercept) 34.36565 6.021081 11 5.707556 0.0001

HerbivoryH -34.36565 7.512579 8 -4.574415 0.0018**

NutrientN -16.86565 7.512579 8 -2.244989 0.0550

SeasonJune -16.66667 7.011984 11 -2.376883 0.0367*

HerbivoryH:NutrientN 17.24065 10.208480 8 1.688856 0.1297

HerbivoryH:SeasonJune 17.04167 9.275983 11 1.837182 0.0933

NutrientN:SeasonJune 13.54167 9.275983 11 1.459863 0.1723

HerbivoryH:NutrientN:SeasonJune -12.91667 12.641033 11 -1.021805 0.3288

Abundance of articulated calcareous algae of established communities. Split plot ANOVA

Value Std.Error DF t-value p-value

(Intercept) 0.00000 10.58612 11 0.0000000 1.0000

HerbivoryH 0.00000 14.00412 8 0.0000000 1.0000

NutrientN 42.50000 14.00412 8 3.0348206 0.0162*

SeasonJune 6.66667 14.78006 11 0.4510582 0.6607

HerbivoryH:NutrientN -42.50000 19.08440 8 -2.2269495 0.0566

HerbivoryH:SeasonJune -6.66667 19.55218 11 -0.3409679 0.7396

NutrientN:SeasonJune -14.79167 19.55218 11 -0.7565226 0.4652

HerbivoryH:NutrientN:SeasonJune 14.79167 26.64513 11 0.5551358 0.5899

Abundance of crustose algae of established communities. Split plot ANOVA

Value Std.Error DF t-value p-value

(Intercept) 5.833333 3.192132 11 1.8274095 0.0949

HerbivoryH -5.833333 4.222794 8 -1.3813918 0.2045

NutrientN -0.833333 4.222794 8 -0.1973417 0.8485

SeasonJune 7.500000 4.514357 11 1.6613662 0.1248

HerbivoryH:NutrientN 0.833333 5.754699 8 0.1448092 0.8884

HerbivoryH:SeasonJune 1.250000 5.971933 11 0.2093125 0.8380

NutrientN:SeasonJune -7.250000 5.971933 11 -1.2140123 0.2502

HerbivoryH:NutrientN:SeasonJune 7.875000 8.138373 11 0.9676381 0.3540
